# Detection, distribution, and functions of RNA *N* ^6^-methyladenosine (m^6^A) in plant development and environmental signal responses

**DOI:** 10.3389/fpls.2024.1429011

**Published:** 2024-07-16

**Authors:** Yang Xiang, Dian Zhang, Lei Li, Yi-Xuan Xue, Chao-Yang Zhang, Qing-Feng Meng, Jin Wang, Xiao-Li Tan, Yu-Long Li

**Affiliations:** School of Life Sciences, Jiangsu University, Zhenjiang, China

**Keywords:** *N* ^6^-methyladenosine (m^6^A), m^6^A sequencing, conserved m^6^A motifs, m^6^A-related proteins, m^6^A functions

## Abstract

The epitranscriptomic mark *N* ^6^-methyladenosine (m^6^A) is the most common type of messenger RNA (mRNA) post-transcriptional modification in eukaryotes. With the discovery of the demethylase FTO (FAT MASS AND OBESITY-ASSOCIATED PROTEIN) in *Homo Sapiens*, this modification has been proven to be dynamically reversible. With technological advances, research on m^6^A modification in plants also rapidly developed. m^6^A modification is widely distributed in plants, which is usually enriched near the stop codons and 3′-UTRs, and has conserved modification sequences. The related proteins of m^6^A modification mainly consist of three components: methyltransferases (writers), demethylases (erasers), and reading proteins (readers). m^6^A modification mainly regulates the growth and development of plants by modulating the RNA metabolic processes and playing an important role in their responses to environmental signals. In this review, we briefly outline the development of m^6^A modification detection techniques; comparatively analyze the distribution characteristics of m^6^A in plants; summarize the methyltransferases, demethylases, and binding proteins related to m^6^A; elaborate on how m^6^A modification functions in plant growth, development, and response to environmental signals; and provide a summary and outlook on the research of m^6^A in plants.

## Introduction

During gene expression, various chemical modifications occur at the levels of DNA, RNA, and proteins. These chemical modifications can preserve genetic information through mechanisms such as DNA and RNA methylation and chromatin conformation changes, all without changing the base sequence (Kumar and Mohapatra, 2021). RNA plays a crucial bridging role in gene expression, and numerous chemical modifications occur on RNA, with more than 170 types discovered so far (Ramakrishnan et al., 2022). These modifications primarily include *N* ^6^-methyladenosine (m^6^A), 5-methylcytidine (m^5^C), 7-methylguanosine (m^7^G), 1-methyladenosine (m^1^A), pseudouridine (Ψ), *N* ^4^-acetylcytidine (ac^4^C), 2′-*O*-methylation (2′-*O*-methyltransferase, Nm-MTase), and *N* ^6^,2′-*O*-dimethyladenosine (m^6^Am), among others (Amos and Korn, 1958; Dunn, 1961; Desrosiers et al., 1974; Stern and Schulman, 1978; Rebane et al., 2002; Luo et al., 2022; Wu et al., 2023). Among them, m^6^A is one of the most abundant chemical modifications on eukaryotic messenger RNA (mRNA), found throughout fungi, animals, and plants (Fu et al., 2014; Deng et al., 2015; Sergiev et al., 2016). Furthermore, m^6^A modification is distributed across various cellular organelles (nucleus, chloroplasts, and the mitochondria) and RNAs (mRNA, non-coding RNA, rRNA, and tRNA) (Cohn and Volkin, 1951; Wang et al., 2017b; Murik et al., 2020). Studies have revealed that m^6^A modification often occurs on the specific motif RRACH (R=G/A, G>A; H=A, C, U), while m^6^A modification in plants also appears on the conserved motif URUAY (Y=A, G, U, or C) (Deng et al., 2018; Cheng et al., 2021). Similar to DNA and histone chemical modifications, m^6^A modification is also dynamically reversible and can be regulated in time and space by methyltransferases and demethylases (Jia et al., 2011). Existing studies have demonstrated that m^6^A modification is involved in the entire growth and development processes, from seed germination to senescence (Rudy et al., 2022; Hu et al., 2022a; Song et al., 2023; Luo et al., 2024). This article systematically reviews the advancement of m^6^A sequencing techniques; the distribution characteristics of m^6^A modification in plants; the m^6^A-related regulatory proteins; and the vital roles of m^6^A in plant growth, development, and response to environmental signals.

## Development of m^6^A modification detection techniques

The modification of m^6^A was first discovered in the mRNA of mammalian cells in the 1970s (Desrosiers et al., 1974). Subsequently, it was also found in plants, such as wheat and corn (Kennedy and Lane, 1979; Nichols, 1979). However, due to technical limitations, m^6^A modification did not receive much attention for many years. Initially, researchers could only detect m^6^A in the hydrolysis products of RNA, without the ability to identify which specific RNA the m^6^A modification originated. Furthermore, limitations in the purification methods made it difficult to exclude the possibility of contamination by RNA types other than mRNA, leading to inaccuracies in the detection of m^6^A (Meyer and Jaffrey, 2017). Another challenge was that, as m^6^A does not affect the binding ability of adenosine to thymine or uracil, it could not be readily detected using conventional hybridization or sequencing-related methods. Instead, its detection relies on specific ribonuclease digestion and chromatographic analysis techniques (Schibler et al., 1977; Zhong et al., 2008; Meyer et al., 2012).

**Figure 1:**
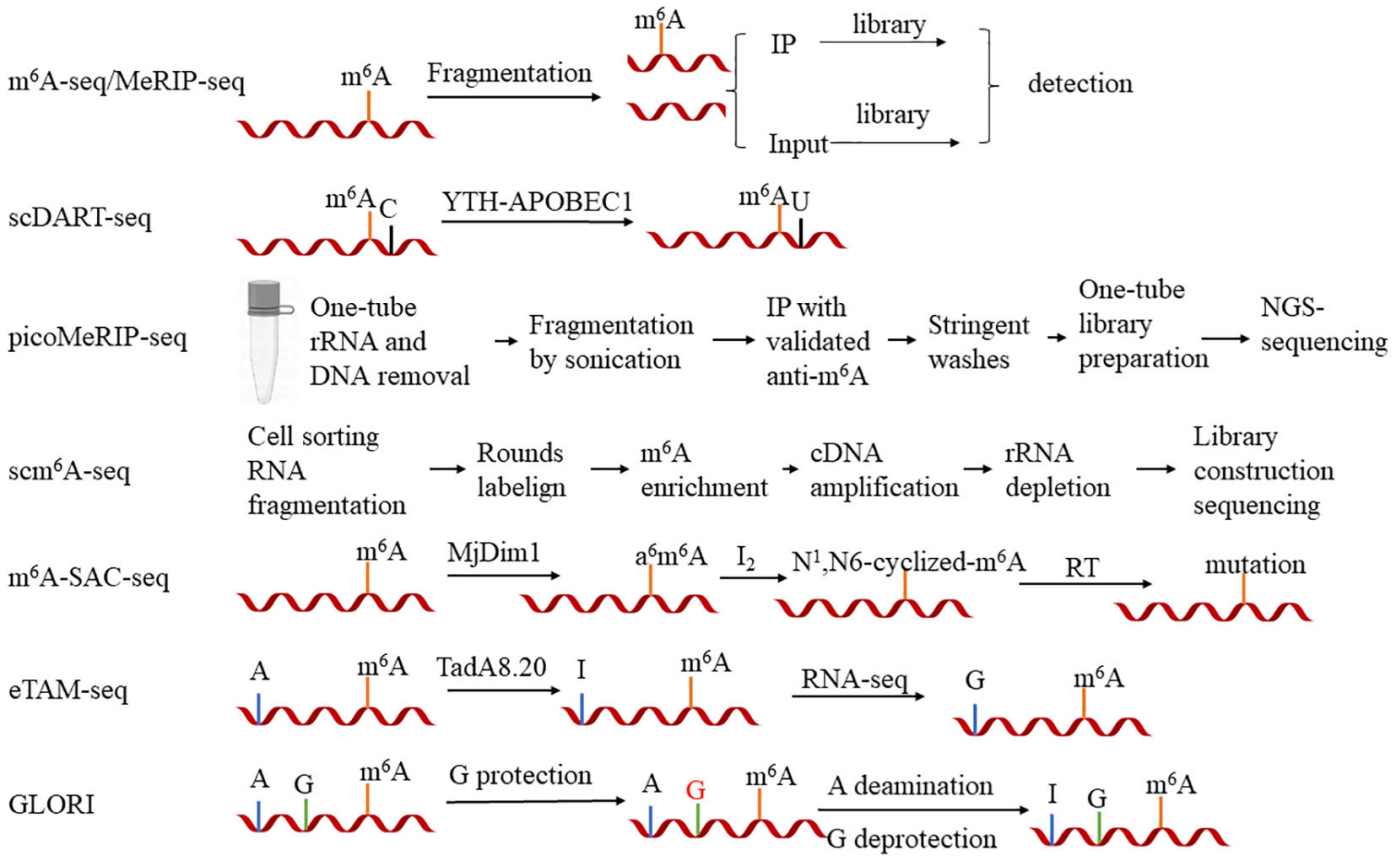
The primary steps of seven major high-throughput sequencing methods for *N* ^6^-methyladenosine (m^6^A) research. 1) m^6^A-seq/MeRIP-seq: Initially, the RNA is fragmented, and m^6^A-modified RNA is enriched using antibodies (*IP*) and without antibodies (*Input*). Libraries are constructed for both groups and compared. Differences indicate the regions containing m^6^A modifications. 2) m^6^A-SAC-seq: m^6^A modifications are incorporated into MjDim1, which is then enzymatically converted to a^6^m^6^A. This is followed by a reaction with iodine monochloride to form *N* ^1^,*N* ^6^-cyclized m^6^A. Detection of the mutated sites through reverse transcription and sequencing can help identify the m^6^A sites. 3) eTAM-seq: The addition of TadA8.20 to RNA can deaminate regular adenine to inosine, while adenine modified with m^6^A is resistant to conversion by TadA8.20. Sequencing identifies the unconverted adenine, thus recognizing the m^6^A modification sites. 4) GLORI: Firstly, guanosine is protected, and then nitrous acid is used to deaminate regular adenine to inosine, with m^6^A remaining unreactive. Subsequently, the protection on guanosine is removed, and sequencing reads the m^6^A sites that did not participate in the reaction. 5) scDART-seq: The YTH-APOBEC1 protein recognizes the cytosines adjacent to the m^6^A sites and deaminates them to uracil. Sequencing detects the signal of cytosine to uracil conversion, identifying the m^6^A sites. 6) picoMeRIP-seq: A single cell is placed in a microtube to remove rRNA and DNA, and RNA fragments are generated by sonication. Subsequently, antibodies are used to enrich the m^6^A-modified RNA and the RNA without antibodies, followed by the elution of RNA with sodium salt. Finally, libraries are constructed in microtubes and sequencing identifies the differential peaks. 7) scm^6^A-seq: Cells are distributed into a 96-well plate and RNA is fragmented. This is followed by two rounds of labeling. Subsequently, the RNA is pooled into a single tube for m^6^A-seq.

The study of m^6^A modification entered a new era following the discovery of the first RNA demethylase, the FAT MASS AND OBESITY-ASSOCIATED PROTEIN (FTO), when it became evident that the modification of m^6^A is dynamically reversible. The field of m^6^A has emerged as a prominent research focus, and the technology for its detection has been rapidly and iteratively updated (Jia et al., 2011). In 2012, two research groups independently introduced a novel m^6^A sequencing technique, known as m^6^A-seq or MeRIP-seq (methylated RNA immunoprecipitation sequencing) (Dominissini et al., 2012; Meyer et al., 2012). The primary steps involve fragmenting RNA into 100- to 200-bp segments, enriching those fragments containing m^6^A using a specific antibody, and subsequently performing reverse transcription sequencing to obtain the sequences of the RNAs harboring m^6^A. This technique revolutionized the research on m^6^A by enabling high-throughput sequencing, propelling the study of m^6^A modification into a new era of rapid development. In recent years, updated sequencing techniques have primarily focused on two areas of optimization: firstly, reducing the initial input of RNA and, secondly, enhancing the resolution of m^6^A detection. Here, we describe several representative methods and their brief steps (**Figure 1**).

In terms of reducing the amount of sample inputs, there are several ways to optimize the sequencing technology. scDART-seq (single-cell deamination adjacent to RNA modification target sequencing) utilizes the APOBEC1 and YTH complex to convert the cytidines adjacent to m^6^A into uridines. Sequencing detects the transition of cytidine to uridine, thereby pinpointing the m^6^A sites (Tegowski et al., 2022). Although this technique can detect m^6^A modification at the single-cell level, it cannot identify the m^6^A sites lacking adjacent cytidines. picoMeRIP-seq (picogram-scale m^6^A RNA immunoprecipitation and sequencing) has optimized MeRIP-seq in areas such as cell lysis strategy, RNA fragmentation method, and RNA elution conditions post-antibody binding, rendering it suitable for the detection of RNA modification with low starting amounts of RNAs/cells (Li et al., 2023b). scm^6^A-seq (single-cell m^6^A sequencing) combines a multiplex labeling approach with the MeRIP-seq principles, enabling concurrent transcriptome and m^6^A methylome sequencing within a single cell. This approach significantly diminishes the initial RNA input and enables sequencing at the single-cell level (Yao et al., 2023a).

In terms of enhancing the resolution of m^6^A detection, numerous novel techniques have emerged. The PA-m^6^A-seq (photo-cross-linking-assisted m^6^A sequencing) strategy initially treats samples with 4-thiouridine (4sU), incorporating 4sU into the RNA samples (4sU induces thymine-to-cytidine mutations at the cross-linking sites). Subsequently, the sample is incubated with an m^6^A antibody to bind to the full-length RNA containing 4sU. UV light at 365 nm is then utilized to induce the cross-linking between the RNA labeled with 4sU and containing m^6^A and the m^6^A antibody. Following this, RNase T1 is employed to digest the RNA into fragments of approximately 30 bp, which are subsequently sequenced (Chen et al., 2015). This method enhances the resolution of m^6^A-seq from 100–200 bp to approximately 30 bp. However, this technique may overlook the m^6^A modifications proximal to the 4sU incorporation sites. m^6^A-SAC-seq (m^6^A selective allyl chemical labeling and sequencing) utilizes an enzymatic reaction to convert m^6^A to a^6^m^6^A. This is further reacted with iodine and then reverse transcribed. During the reverse transcription, m^6^A is interpreted as a mutant base, enabling the detection of the m^6^A sites as mutant bases through sequencing (Hu et al., 2022c; Ge et al., 2023). This reaction employs an enzymatic method for detection and may exhibit uncertain sequence preferences. Another quantitative technique is eTAM-seq (evolved TadA-assisted *N* ^6^-methyladenosine sequencing), which utilizes the deaminase TadA8.20 to convert normal adenosines into inosines. During sequencing, the inosines are misread as guanosines, whereas m^6^A remains unchanged and is still interpreted as adenosine. Through this process, the m^6^A sites can be identified. Notably, eTAM-seq exhibits reduced sensitivity to sites with low methylation levels (Xiao et al., 2023). The GLORI (glyoxal- and nitrite-mediated deamination of unmethylated adenosines) technique employs a system catalyzed by glyoxal and nitrite salts to efficiently deaminate the unmethylated adenosines into inosines (A-to-I, >98%). During sequencing, the inosines are interpreted as guanosines (G), resulting in A-to-G conversions. m^6^A, however, is still read as an adenosine. GLORI achieves absolute quantification of m^6^A at single nucleotide resolution by analyzing the proportion of adenosines in the sequence reads. One drawback of GLORI is its relatively high sequencing cost compared with that of MeRIP or m^6^A-seq (Liu et al., 2023).

With the rapid development of m^6^A sequencing technologies, the accuracy of sequencing has continuously improved and the demand for RNA samples has also significantly decreased. Essentially, highly efficient, highly sensitive, highly specific, and unbiased single-nucleotide m^6^A site detection has been achieved. The progress in detection technologies has also greatly propelled the research into m^6^A modification. The distribution characteristics and the biological functions of m^6^A have been rapidly revealed.

## Distribution of m^6^A in plants

m^6^A is widely found in fungi, animals, and plants (Deng et al., 2015; Sergiev et al., 2016). In plants, m^6^A modification is distributed across the start codon, the stop codon, the coding sequence (CDS), and the 5′-UTR and 3′-UTR of mRNAs. However, this distribution is not random, and different species exhibit distinct tendencies and patterns (**Table 1**). In *Arabidopsis*, rice, tomato, maize, tea tree, wolfberry, and sea buckthorn, m^6^A modifications are primarily enriched around the stop codon and 3′-UTR regions (Wan et al., 2015; Du et al., 2020; Cheng et al., 2021; Zhang et al., 2021a; Hu et al., 2022a; Zhao et al., 2023; Zhu et al., 2023). In watermelon and apple, m^6^A modifications are enriched in the CDS (Mao et al., 2021; Hou et al., 2022). Notably, in *Arabidopsis*, pear, rice, and soybean, there is a tendency for m^6^A modifications to be enriched around the start codon as well (Luo et al., 2014; Li et al., 2014b; Han et al., 2021; Zhang et al., 2023a). In coastal pine, m^6^A modifications are enriched in the 5′-UTR region (Ortigosa et al., 2022).

**Table 1:** Distribution and conserved motifs of *N* ^6^-methyladenosine (m^6^A) modification in different plants.

Plant species	Distribution sites	Conserved motifs
Arabidopsis thaliana	initiation codon, stop codon, 3`UTR	RRACH, URUAY
Triticum aestivum L. Oryza sativa L.	stop codon , 3`UTR stop codon , 3`UTR	RRACH, URUAY RRACH,URUAY, UGWAMH, DGGACU
Solanum lycopersicum L	stop codon , 3`UTR	RRACH, URUAY
Citrullus lanatus Pyrus spp	3`UTR, CDS CDS, initiation codon, stop codon	RRACH, URUAY RRACH, URUAY
Gossypium spp Zea mays L. Malus pumila Mill.	3`UTR stop codon, 3`UTR 3`UTR, CDS	RRACH, URUAY, DGCAG RRACH, URUAY URUAY

R, adenosine or guanosine; H, adenosine, cytidine, and uridine; M, adenosine or cytidine; D, adenosine, guanosine, or uridine; W, adenosine or uridine; CDS, coding sequence; UTR, untranslated region.

The conserved m^6^A sequence motif in plants is identical to that in eukaryotes, being RRACH (R=G or A; H=A, C, or U) (Meyer et al., 2012). However, recent studies have identified plant-specific conserved sequences, such as URUAY (Y=A, G, U, or C), which has been detected in *Arabidopsis*, rice, wheat, tomato, maize, tea tree, wolfberry, and cotton (Wan et al., 2015; Zhou et al., 2019; Du et al., 2020; Zhu et al., 2021; Zhang et al., 2021b, d; Zhao et al., 2023; Li et al., 2023a). In addition, rice exhibits specific conserved sequences, including UGWAMH (W=U or A; M=C or A; H=U, A, or C), CGVCGRC (V=A/C/G; R=A/G), and DGGACU (D=A/G/U) (Zhang et al., 2019; Wang et al., 2022c). In *Chlamydomonas reinhardtii* mRNA, m^6^A modifications predominantly occur within the conserved sequence DRAC (D=G/A/U; R=A/G) (Lv et al., 2022). Furthermore, cotton has recently revealed conserved sequences such as DGCAG (D=A/G/U) and the 5′-UTR enriched sequence CAAUG (Li et al., 2023a).

## m^6^A modification-related proteins

**Figure 2:**
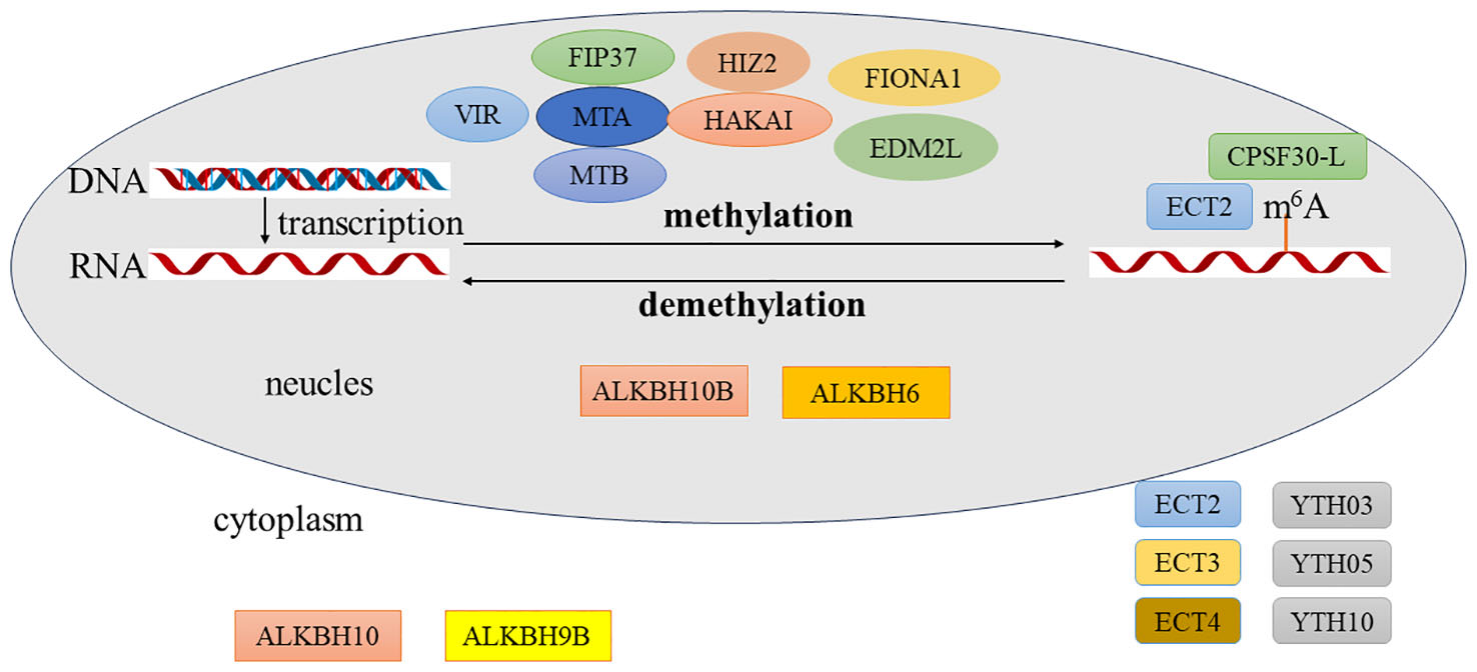
Process of *N* ^6^-methyladenosine (m^6^A) modification in plants. The methyltransferases involved in m^6^A modification include FIP37, MTA, MTB, VIR, HIZ2, and EDM2. The demethylases include ALKBH10, ALKBH9B, and ALKBH6. The reading proteins include ECT2, ECT3, ECT4, YTH03, YTH04, YTH05, and CPSF30-L. The methyltransferases for m^6^A modification are all located in the cell nucleus. The demethylase ALKBH10B is found in both the cell nucleus and the cytoplasm, ALKBH6 is located in the cell nucleus, and ALKBH9B is in the cytoplasm. The reading protein ECT2 is located in both the cell nucleus and the cytoplasm, CPSF30-L is in the cell nucleus, and the rest are in the cytoplasm.

The modification of m^6^A methylation, akin to chemical modifications of DNA and histone, is also dynamic and reversible. This process involves a complex of methyltransferases, reading proteins, and demethylases, which are responsible for writing, reading, and erasing the modification, respectively (**Figure 2**).

### Writers

In mammals, the m^6^A methyltransferase complex (also known as the m^6^A writer) is composed of METTL3, METTL14, WTAP, and other proteins. METTL3 and METTL14 can form a heterodimer, and WTAP interacts with this dimer to achieve methylation (Liu et al., 2014; Ping et al., 2014). In addition, researchers successively discovered several enzymes involved in the methylation of m^6^A: METTL16, VIRMA (vir-like m^6^A methyltransferase associated), HAKAI (E3 ubiquitin-protein ligase Hakai), RBM15 (RNA-binding motif 15), and ZC3H13 (zinc finger CCCH domain-containing protein 13) (Horiuchi et al., 2013; Liu et al., 2014; Ping et al., 2014; Schwartz et al., 2014; Patil et al., 2016; Wen et al., 2018). These enzymes are collectively referred to as the m^6^A/METTL-associated complex (MACOM) (Knuckles et al., 2018).

In plants, researchers discovered the methyltransferase component MTA (methyltransferase A) in *Arabidopsis*. MTA is a homolog of METTL3 and participates in plant embryo development (Luo et al., 2024). They also found its interacting protein, FIP37 (FKBP12 interacting protein, 37 kDa) (Zhong et al., 2008). In fact, FIP37 was discovered in *Arabidopsis* as early as 2004 and was found to regulate embryo development. Subsequent research revealed that FIP37 is also a methyltransferase component and a homolog of WTAP (Vespa et al., 2004; Shen et al., 2016). In addition, MTB is the plant homolog of METTL14. VIR (virilizer) is the plant homolog of VIRMA, and the plant homolog of HAKAI is also named HAKAI (Růžička et al., 2017). HIZ2 (HAKAI-interacting zinc finger protein 2) is the plant homolog of ZC3H13, which has been found to be associated with lateral root formation in *Arabidopsis* (Zhang et al., 2022a). FIONA1, the plant homolog of METTL16, was discovered by several research groups to be related to flowering, chlorophyll homeostasis, and salt stress in *Arabidopsis* (Sun et al., 2022; Xu et al., 2022; Wang et al., 2022a; Jiang et al., 2023; Cai et al., 2024a). Currently, no homolog of RBM15 has been found in plants. Furthermore, a plant-specific m^6^A methyltransferase, EDM2L, was discovered in rice, which participates in the regulation of pollen development (Ma et al., 2021).

Like in animals, the m^6^A methyltransferases in plants exist in complex form and interact with each other. As for the five methyltransferase components in *Arabidopsis*—FIP37, MTA, MTB, VIR, and HAKAI—they do not significantly affect each other at the transcriptional level. However, at the protein level, these components mutually influence each other, promoting each other’s protein accumulation, and they cannot functionally substitute for one another (Shen, 2023).

### Erasers

The methylation can be removed by demethylases, which are known as m^6^A erasers. In 2011, two mammalian m^6^A demethylases, i.e., FTO and ALKBH5, were discovered successively (Jia et al., 2011; Zheng et al., 2013). These enzymes belong to the divalent iron and α-ketoglutarate-dependent dioxygenase AlkB family. They initially oxidize m^6^A to form *N* ^6^-hydroxymethyladenosine (hm^6^A), subsequently convert hm^6^A to *N* ^6^-formyladenosine (f^6^A), and ultimately transform f^6^A into adenosine (A), thus completing the demethylation process (Wang et al., 2020). Nine proteins belonging to the AlkB family have been discovered in humans, including ALKBH1–8 and FTO. The identification of m^6^A demethylases in plants is highly significant, as it would directly demonstrate that m^6^A plays a dynamic and reversible regulatory role in plants. While no FTO homologs have been found in plants, there are 13 homologs of the AlkB family in *Arabidopsis* (Mielecki et al., 2012). Of these, AtALKBH10B, AtALKBH6, and AtALKBH9C have been identified as possessing demethylation functions (Duan et al., 2017; Huong et al., 2020; Amara et al., 2022). Furthermore, CsALKBH4 in tea, CfALKBH5 in *Catalpa fargesii*, SlALKBH2 and SlALKBH10B in tomato, GhALKBH10 and GhALKBH10B in cotton, OsALKBH9 in rice, PagALKBH9B and PagALKBH10B in *Populus*, LbALKBH10 in wolfberry, and HrALKBH10B, HrALKBH10C, and HrALKBH10D in sea buckthorn have also been identified as having demethylation functions (Zhou et al., 2019; Zhang et al., 2021a; Cui et al., 2022; Zhao et al., 2022, 2023; Zhu et al., 2023; Li et al., 2023a; Shen et al., 2023a; Zhang et al., 2023c; Tang et al., 2024). Given the numerous AlkB family homologs in plants and the diverse functions of the AlkB family proteins, the identification of more m^6^A demethylases requires extensive and detailed research.

### Readers

Methylation can be recognized by m^6^A-binding proteins, known as m^6^A readers. Knocking out or overexpressing methyltransferases and demethylases results in various phenotypes caused by changes in the m^6^A levels, providing proof that m^6^A plays an important role in biological growth and development processes. However, to understand the specific molecular mechanism by which m^6^A functions, it is crucial to determine how the m^6^A reader proteins operate. Most of the currently discovered m^6^A reading proteins contain the YTH (YT512-B homology) structural domain. Initially, the YTH structural domain was only regarded as an ordinary RNA-binding structural domain (Zhang et al., 2010). Subsequently, it was discovered that this structural domain recognizes m^6^A modifications (Wang et al., 2014a). The YTH structural domain contains a hydrophobic functional domain composed of aromatic amino acid residues, which enhances the affinity of the reading protein for m^6^A, allowing the protein to recognize m^6^A modifications (Luo and Tong, 2014; Arribas-Hernández et al., 2020).

The YTH structural domain family proteins constitute a highly conserved protein family in eukaryotic cells. Bioinformatics analysis has revealed the existence of YTH family proteins in humans, mice, fruit flies, yeast, *Arabidopsis*, and rice, with plants being particularly abundant in them. In *Arabidopsis*, the YTH structural domain is called the evolutionarily conserved C-terminal region (ECT) domain, encompassing a total of 13 members, named ECT1–ECT12 and CPSF30 (Ok et al., 2005; Li et al., 2014a). Of these, ECT2 was the first m^6^A reader protein discovered in plants that possesses the YTH structural domain. ECT2 binding sites are strongly enriched in the 3′-UTRs of target genes, and their function is tied to trichome morphology (Wei et al., 2018). Subsequent studies conducted by the same laboratory revealed that, in *Arabidopsis*, ECT2, ECT3, and ECT4 directly interact with each other in the cytoplasm and perform genetically redundant functions in the regulation of abscisic acid (ABA) response during seed germination and post-germination growth (Song et al., 2023). In addition, ECT2, ECT3, and ECT4 are also involved in normal leaf morphogenesis and the rate of leaf formation (Arribas-Hernández et al., 2020). ECT8 serves as a crucial checkpoint for the negative feedback regulation of ABA signaling by sequestering the m^6^A-modified ABA receptor gene PYRABACTIN RESISTANCE 1-LIKE 7 (*PYL7*) through phase-separated ECT8 condensates in stress granules in response to ABA (Wu et al., 2024). In *Arabidopsis*, the *AtCPSF30* (30-kDa cleavage and polyadenylation specificity factor 30) gene encodes two differently sized proteins, CPSF30-S and CPSF30-L, via alternative polyadenylation (APA) regulation after transcription. CPSF30-L comprises CPSF30-S and an m^6^A-binding YTH domain (Hou et al., 2021). CPSF30-L, as an *Arabidopsis* m^6^A reader, requires its m^6^A-binding function for floral transition and ABA response (Song et al., 2021). Moreover, ECT1 can be recruited to ECT9 condensates and plays a negative role in plant immunity (Wang et al., 2023a). ECT12 binds to m^6^A-modified stress-responsive transcripts and plays a crucial role in the response to salt or dehydration stress (Amara et al., 2024). In rice, YTH03, YTH05, and YTH10 specifically bind to m^6^A-containing RNAs and regulate the plant height of rice in a functionally redundant manner. Furthermore, YTH07 can physically interact with EHD6, and it triggers the relocation of a portion of YTH07 from the cytoplasm into RNP granules through phase-separated condensation, leading to accelerated flowering (Cui et al., 2024). In apple, the YTH domain-containing RNA-binding protein 1 (MhYTP1) and MhYTP2 have functions in leaf senescence and fruit ripening and confer tolerance to multiple abiotic stresses (Wang et al., 2017a).

In addition, the insulin-like growth factor 2 mRNA-binding proteins (IGF2BPs), which contain four KH domains but lack YTH domains, have been demonstrated to form a distinct family of m^6^A readers that recognize the consensus motif GG(m^6^A)C (Huang et al., 2018). Studies have found that IGF2BPs are associated with mRNA stability and tumorigenesis (Ying et al., 2021). Based on amino acid sequence similarity, FLK was identified as an *Arabidopsis* homolog of IGF2BP, regulating floral transition by repressing the levels of a key floral repressor, FLOWERING LOCUS C (FLC), in *Arabidopsis*. FLK directly binds to the FLC mRNA and regulates the expression of FLC in an m^6^A-dependent manner (Amara et al., 2023).

## Biological functions of m^6^A

As the most abundant internal modification on mRNA in eukaryotes, m^6^A can affect various RNA metabolic processes, including mRNA stability, precursor RNA splicing, polyadenylation, mRNA transport, and translation initiation (Zaccara et al., 2019). In human and animal cells, m^6^A modification participates in important physiological processes, such as tumor and cardiovascular disease development and osteocyte differentiation (Huang et al., 2021). Although research on the modification of m^6^A in plants started relatively late, numerous studies in recent years have demonstrated that it plays a crucial role in plant growth and development, biotic and abiotic stress responses, and crop trait improvement (Shao et al., 2021; Shen et al., 2023b). In particular, when plants are subjected to external environmental stresses, the dynamic and reversible changes in m^6^A modification can rapidly regulate gene expression, thereby conferring strong environmental adaptability to plants (Hu et al., 2022b).

### m^6^A affects mRNA metabolism

In mammalian cells, numerous studies have demonstrated that m^6^A modification is linked to mRNA metabolism (Wang et al., 2014b). With the increasing research interest in plant m^6^A in recent years, it has also been confirmed that m^6^A is linked to RNA metabolism in plants. Studies have found that following the mutation of *AtMTA*, the level of m^6^A decreases, and the rate of degradation of the mRNA encoding the core component of the molecular oscillator circadian clock associated 1 (CCA1) accelerates (Wang et al., 2021). Research has also shown that the disruption of ALKBH10B elevates the m^6^A modification levels of FT, SPL3, and SPL9 mRNAs, accelerating their degradation (Duan et al., 2017). Moreover, the CPSF30-L protein primarily recognizes the m^6^A-modified far-upstream elements to control the choice of the polyadenylation site, lengthens the 3′-UTRs of transcripts, and thereby accelerates their mRNA degradation (Hou et al., 2021; Song et al., 2021). Another study discovered that R-loops are structures formed by the hybridization of RNA and DNA and that m^6^A modification in *Arabidopsis* can affect the strength of R-loops and promote gene transcription (Thomas et al., 1976; Zhang et al., 2021c). Furthermore, m^6^A modification can stabilize mRNA by inhibiting the cleavage action of local ribonucleases (Anderson et al., 2018). In *Arabidopsis*, the reader protein ECT12 plays a crucial role in modulating the stability of the m^6^A-marked RNA transcripts, thereby enhancing the ability of plants to cope with abiotic stresses, such as salt and drought (Amara et al., 2024). The regulation of mRNA stability by FIONA1-mediated m^6^A methylation also influences the expression of the genes involved in salt stress response (Cai et al., 2024a). On the other hand, as an “eraser,” ALKBH10B contributes to drought resistance by promoting the stability of transcripts, and the impact of OsALKBH9 on pollen development is also linked to its mediation of mRNA stability (Han et al., 2023a; Tang et al., 2024).

Current research indicates that there is also an important connection between the location of the m^6^A modification sites on mRNA and mRNA stability. Generally, m^6^A in the 3′-UTR tends to be negatively correlated with the gene expression levels, while m^6^A at the 5′ end is positively correlated with gene expression. VIR affects the elongation of 3′-UTR through alternative polyadenylation, thereby negatively regulating the mRNA stability of several salt stress-negative regulators and modulating the homeostasis of reactive oxygen species (ROS) (Hu et al., 2021). In strawberry, m^6^A modification in CDS regions appears to be ripening-specific and tends to stabilize the mRNAs, whereas m^6^A around the stop codons and within the 3′-UTRs is generally negatively correlated with the abundance of associated mRNAs. FLK, as an m^6^A reader protein, directly binds to a site in the 3′-UTR of FLC transcripts, repressing the FLC levels by reducing its stability and splicing (Amara et al., 2023).

m^6^A modification is also involved in mRNA translation and alternative splicing. In wheat, the m^6^A in the CDS and 3′-UTR inhibits mRNA translation, while that in the 5′-UTR and start codon can promote mRNA translation (Huang et al., 2022a). In *Arabidopsis*, FIONA1 regulates the accuracy and efficiency of U6 snRNA splicing (Parker et al., 2022). Genes with methylation in the 3′-UTR in soybean have higher expression levels and are more prone to alternative splicing (Zhang et al., 2023a). FLK directly binds to the m^6^A sites in the 3′-UTR of FLC transcripts, suppressing the levels of FLC by decreasing the transcript stability and splicing (Guo et al., 2022; Amara et al., 2023). The rice EDM2L altered the transcriptomic m^6^A landscape and caused a distinct m^6^A modification of the EAT1 transcript, leading to the dysregulation of its alternative splicing and to polyadenylation. This, in turn, affects anther development in rice (Ma et al., 2021).

### Role of m^6^A in plant growth and development

**Figure 3:**
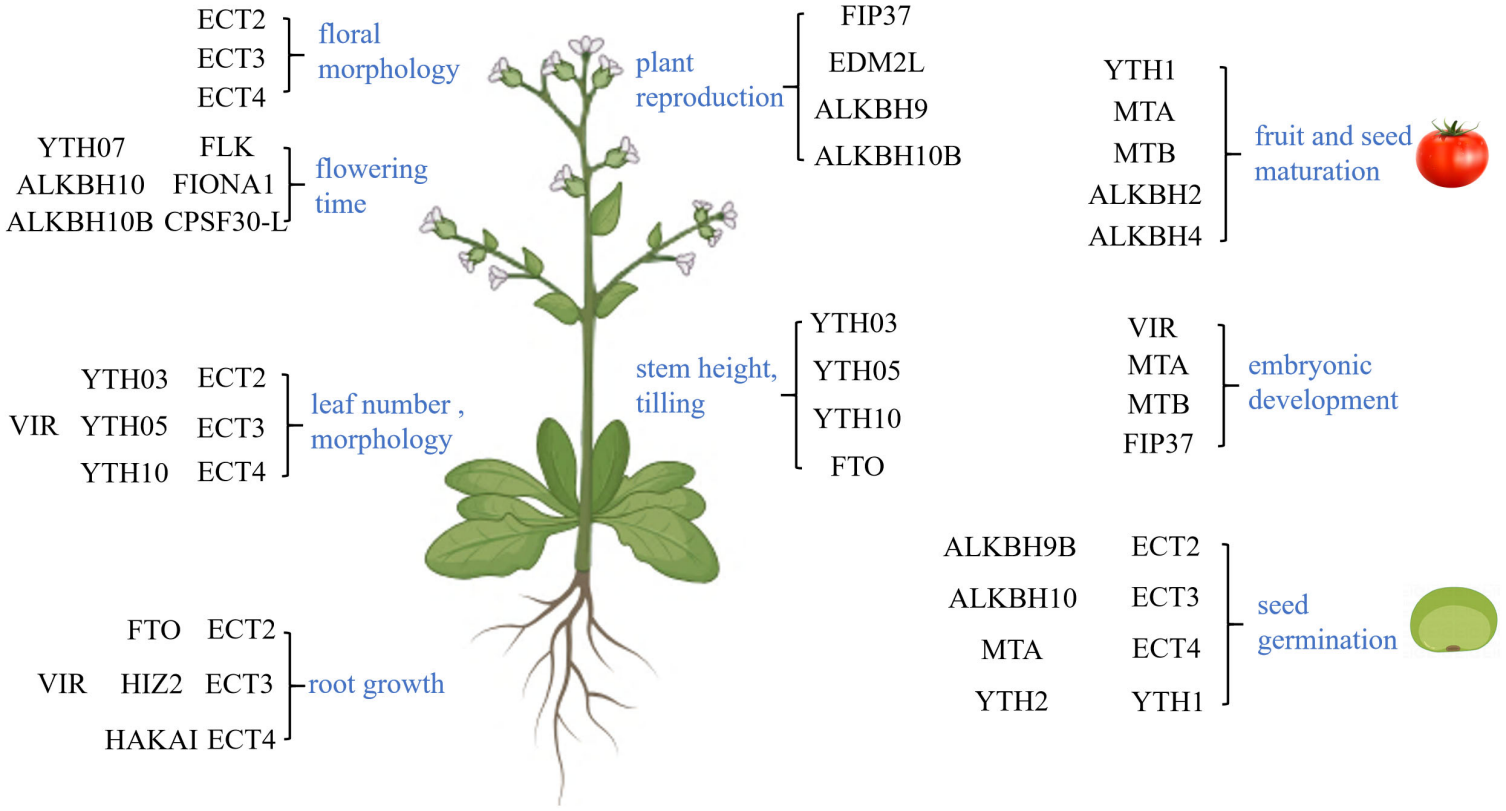
Function of *N* ^6^-methyladenosine (m^6^A) in plant growth and development. m^6^A primarily regulates the flower morphology, flowering time, leaf number and morphology, root growth, plant reproduction, stem height, tilling, fruit and seed maturation, embryonic development, and seed germination, which are marked in *blue*. These functions are primarily achieved through its associated proteins.

m^6^A plays a crucial role throughout the plant life cycle, encompassing seed germination, embryo development, and root, stem, and leaf growth. This is in addition to its involvement in flowering and fruit maturation, all of which rely on precise regulation through m^6^A modification (**Figure 3**).

m^6^A modification can influence floral morphology. Research has demonstrated that *ect2/ect3/ect4* mutants exhibit a slower flower formation and an aberrant flower morphology (Arribas-Hernández et al., 2020). Furthermore, m^6^A modification regulates the flowering time. FIONA1 regulates floral transition by influencing the splicing of FLC and the stability of the floral activators SPL3 and SEP3 (Sun et al., 2022; Xu et al., 2022; Wang et al., 2022a; Cai et al., 2023a). In addition, the ALKBH10B-mediated mRNA demethylation enhances the mRNA stability of FLOWERING LOCUS T (FT), SPL3, and SPL9, thereby regulating flowering (Duan et al., 2017; Liu et al., 2024b). Research has found that FLK directly binds to the m^6^A modification site in the 3′-UTR of FLC, suppressing the levels of FLC by decreasing its stability and splicing, thus regulating flowering (Amara et al., 2023). The m^6^A-binding function of CPSF30-L selects the proximal poly(A) site and generates a short 3′-UTR at SOC1, RPN10, and FYVE1, thereby preventing mRNA degradation and regulating the floral transition and ABA response (Song et al., 2021). Moreover, YTH07 can physically interact with EHD6, which enhances the binding of an m^6^A-modified RNA and triggers the relocation of a portion of YTH07 from the cytoplasm into the RNP granules through phase-separated condensation. This leads to the sequestration of the mRNA of the rice flowering repressor OsCOL4, resulting in a reduction in its protein abundance and, thus, in accelerated flowering in rice (Cui et al., 2024).

m^6^A modification also plays an important role in plant reproductive regulation, primarily by regulating pollen development and affecting the plant reproductive process. OsFIP37 mediates the modification of m^6^A on an auxin biosynthesis gene, *OsYUCCA3*, during microsporogenesis, which is essential for meiotic division and subsequent pollen development in rice (Cheng et al., 2022). Moreover, OsEDM2L regulates EAT1 transcription by interacting with bHLH142 and TDR, and it further mediates m^6^A modification, alternative splicing, and polyadenylation of the EAT1 transcripts. This controls the expression of tapetal programmed cell death (PCD)-related genes and, subsequently, male reproduction (Ma et al., 2021). OsALKBH9 reduces the m^6^A modifications in the TDR and GAMYB transcripts and affects their stability to regulate pollen development (Tang et al., 2024). Furthermore, m^6^A RNA methylation impairs the gene expression variability and reproductive thermotolerance in *Arabidopsis*. Disruption of AtALKBH10B leads to a lower gene expression variability, the suppression of heat-activated genes, and a strong reduction in plant fertility (Wang et al., 2022b).

m^6^A modification is also closely related to embryo development. After the inactivation of AtMTA, the reduced level of m^6^A modification leads to the inability of developing embryos to pass through the globular stage (Zhong et al., 2008). ZmMTA dysfunction leads to severe arrest in maize embryogenesis and endosperm development (Cai et al., 2024a). Knocking out the *AtFIP37* gene leads to a strong delay in endosperm development and embryo arrest, resulting in embryo lethality (Vespa et al., 2004). MiR408 activates nucleotide metabolism by inhibiting DlNUDT23, thereby regulating m^6^A modification and ultimately promoting early embryogenesis in longan (Xu et al., 2023b). FIP37-mediated m^6^A modification accelerates the degradation of WUSCHEL (WUS) and SHOOTMERISTEMLESS (STM), limiting their transcript levels to prevent excessive shoot apical meristem (SAM) proliferation (Shen et al., 2016). The inactivation of MTC, MTA, MTB, and FIP37 in moss leads to the loss of m^6^A, resulting in delayed gametophyte bud formation and in defective spore development (Garcias-Morales et al., 2023).

The development of plant roots, stems, and leaves is also regulated by m^6^A modification. In terms of root growth, research has found that by treating Moso bamboo with the RNA methylation inhibitor DZnepA, reducing its m^6^A modification level can increase the number of its lateral roots (Liufu et al., 2023). At the same time, studies have also found that the overexpression of the *HIZ1* gene in *Arabidopsis* leads to an increase in the m^6^A modification levels, thereby reducing lateral roots (Zhang et al., 2022a). In addition, the *ect2/ect3/ect4* mutants exhibit slow root growth and defects in root growth directionality (Arribas-Hernández et al., 2020). The silencing of *GhVIR* reduces the level of m^6^A modification, affecting the cell size, shape, and total cell number of cotton leaves, thereby affecting their morphogenesis (Huang et al., 2022b). In rice, YTH03/05/10 are cytoplasmic proteins that are highly expressed in the stem and leaf sheath (Cai et al., 2023b). Studies have found that loss of the function of YTH03/05/10 leads to dwarfism in rice. Moreover, the heterologous expression of *FTO* can increase the removal of m^6^A modification in rice to stimulate the proliferation of root meristem cells and the formation of tiller buds (Yu et al., 2021).

Seed germination is influenced by many factors, with m^6^A modification emerging as a crucial regulator. This modification primarily impacts seed germination by modulating the ABA response. ECT2 directly interacts with the PAB2 and PAB4 proteins, maintaining the stability of DWA1, DWA2, SDIRIP1, and CPN20 mRNAs. This process promotes the accumulation of ABI5, thereby regulating the ABA-mediated seed germination and the subsequent growth (Song et al., 2023). On the other hand, ALKBH9B negatively regulates the ABA response by reducing m^6^A modification, which leads to the increased mRNA stability of ABA INSENSITIVE 1 (ABI1) and BRI1-EMS-SUPPRESSOR 1 (BES1) during seed germination (Tang et al., 2021, 2022).

m^6^A modification can also regulate the development of fruits. Taking tomato fruit as an example, the fruit gradually increases in size during the transition from immature green to mature red. During this process, the overall m^6^A level and mRNA abundance also increase (Hu et al., 2022a). Studies have shown that, once the m^6^A modification site is recognized, SlYTH1 enhances the stability of the gibberellin (GA)-related genes, ultimately elevating the seed germination rate and promoting fruit development (Yin et al., 2022). SlALKBH2 has the ability to bind the transcript of the DNA demethylase gene *SlDML2*, which is required for tomato fruit ripening, and positively affects fruit ripening by regulating its stability through the demethylation of m^6^A (Zhou et al., 2019). MTA can also affect the ABA response by increasing the m^6^A modification level, enhancing the stability of NCED5 and AREB1 mRNA, or promoting the translation efficiency of ABAR, thereby promoting the maturation of strawberry fruits (Zhou et al., 2021). Furthermore, m^6^A affects the stability of the mRNAs related to fiber elongation, ultimately influencing cotton fiber elongation (Xing et al., 2023). The modification of m^6^A is closely related to the accumulation of substances during fruit maturation. Studies have shown that m^6^A modification significantly increases in the late stage of wheat seed development, particularly enriched in pathways related to protein and starch synthesis, indicating its close association with the accumulation of substances during wheat seed maturation (Li et al., 2022). In addition, not only the maturation of fruits but also the accumulation of substances in plant leaves is related to m^6^A modification. In tea plants, CsALKBH4 affects the stability and the abundance of the transcripts related to terpene biosynthesis by removing m^6^A modification, directly affecting the accumulation of volatile terpene compounds and the aroma of tea leaves. At the same time, by activating selective polyadenylation during sunlight withering, it indirectly regulates the content of flavonoids, catechins, and theaflavins, as well as the formation of substances related to tea flavor (Zhu et al., 2023).

### m^6^A is involved in the plant response to environmental signals

Due to their immobility, plants have evolved a set of mechanisms through long-term natural selection to withstand the surrounding environment. In the process of plants responding to different environments, m^6^A modification also plays an important role.

#### Abiotic environmental signals

Light is an important signal for regulating plant growth and morphological development. Light can affect the morphology of plants through m^6^A modification as well. In *Arabidopsis*, the blue light receptor CRY1 interacts with FIP37, modulating m^6^A on the photomorphogenesis-related genes *PIF3*, *PIF4*, and *PIF5*, thereby accelerating the decay of their transcripts and repressing the elongation of the hypocotyl (Yang et al., 2023). Light can also regulate the circadian rhythm of plants through m^6^A modification. The blue light receptor CRY2 interacts with the mRNA m^6^A methyltransferase complex (MTA/MTB/FIP37), and by increasing the m^6^A modification level, it alters the degradation rate of the mRNA of the core circadian gene *CCA1*, thereby affecting the circadian clock in plants (Wang et al., 2021). Furthermore, the blue light-excited CRY2 undergoes liquid–liquid phase separation (LLPS) to form photobodies that recruit the m^6^A “writer” complex, regulates the methylation of the transcriptome, and is involved in the regulation of chlorophyll homeostasis (Jiang et al., 2023). Seagrass exhibits a peak of m^6^A modification during the dark period under the same photoperiod. The methylation of m6A could widely contribute to circadian regulation in seagrass, potentially affecting the photobiological behavior of these plants (Ruocco et al., 2020).

Salt stress is a major abiotic stress during plant growth. Mutants of *mta*, *mtb*, *vir*, and *hakai* in *Arabidopsis* exhibit m^6^A-dependent salt sensitivity. VIR-mediated m^6^A methylation modulates ROS homeostasis by negatively regulating the mRNA stability of several salt stress-negative regulators, including ATAF1, GI, and GSTU17, through affecting the 3′-UTR lengthening linked to alternative polyadenylation (Hu et al., 2021). AtECT12 promotes greater stabilization of NHX1, a positive regulator of salt stress, and decreases the stability of BGLU22 and GSTU17, which are negative regulators of salt stress, thereby positively regulating salt stress response (Lee et al., 2024). Increased AtECT8 leads to the enhanced binding of m^6^A-modified mRNAs, thereby accelerating the degradation of the negative regulators of salt stress response to enhance salt tolerance (Cai et al., 2024b). PagFIP37 regulates the mRNA stability of the salt-responsive transcripts in an m^6^A manner and plays a positive role in the response of poplar to salt stress (Zhao et al., 2024b). In rice, transcripts encoding the transcription factors, antioxidants, and auxin response-related genes exhibit changes in the m^6^A methylation levels in shoots or roots under salt stress, implying that m^6^A may mediate salt tolerance by regulating transcription, ROS homeostasis, and auxin signaling in a tissue-specific manner (Wang et al., 2022c). AtFIONA1-mediated m^6^A methylation regulates the production of ROS and affects the transcription levels of the salt stress-responsive genes by regulating their mRNA stability (Cai et al., 2024a). Silencing of the *GhALKBH10* gene in cotton can increase the m^6^A modification level, enhance the antioxidant capacity, and reduce the Na^+^ concentration in the cytoplasm, thereby improving the plant’s tolerance to salinity (Cui et al., 2022). SlALKBH10B negatively regulates cell damage in salt stress, thereby rendering plants salt-intolerant (Shen et al., 2023a).

Drought is an environmental condition that plants often face. m6A modification primarily affects plant drought resistance in three ways. Firstly, it enhances plant drought tolerance by regulating the root system. Studies have found that PtrMTA in poplar increases the level of m^6^A modification, promoting root hair density and root growth, thereby improving tolerance to drought stress (Lu et al., 2020). PagALKBH9B and PagALKBH10B in poplar reduce the number of adventitious roots and the accumulation of biomass by decreasing the m^6^A level, leading to the decreased adaptability of plants to drought stress (Zhao et al., 2022). Secondly, m^6^A modification affects the expression of the drought-related genes under drought conditions (Mao et al., 2021). The overexpression of *ClMTB* in tobacco plants increased drought tolerance by enhancing the ROS scavenging system and alleviating photosynthesis inhibition under drought stress through increasing the m^6^A level (He et al., 2021). Studies have also found that SiYTH1 can stabilize SiARDP and the ROS removal-related transcripts SiAPX1, SiGRXC7, and SiGULLO4, thereby promoting stomatal closure and ROS clearance and enhancing drought resistance in *Setaria italica* (Luo et al., 2023). Furthermore, ECT12 and ALKBH10B positively regulate drought resistance by affecting the stability of the mRNAs involved in drought stress response in *Arabidopsis* (Amara et al., 2024). Thirdly, m^6^A modification affects drought resistance by regulating the ABA response. In sea buckthorn, m^6^A modification can regulate the expression levels of the ABA-related genes to enhance resistance to drought stress (Zhang et al., 2021a). GhALKBH10B was found to reduce the level of m^6^A in cotton, leading to the degradation of the mRNAs of the ABA signal-related genes and the Ca^2+^ signal-related genes, which is unfavorable for plant drought resistance (Li et al., 2023a).

Under low-temperature conditions, m^6^A modification can affect the translation efficiency and photosynthetic efficiency. Research has shown that the downregulation of FIP37 has no particular effect on photosynthesis under standard conditions, but is crucial for efficient photosynthesis and other chloroplast functions related to plant growth during cold acclimation (Vicente et al., 2023). Furthermore, m^6^A modification also affects pollen formation under low temperatures. Low-temperature stress leads to a decrease in the overall m^6^A level in tomato anthers, but increases the m^6^A modification of the ATP-binding cassette G31 (SlABCG31) in the ATP-binding pathway, leading to the decreased expression of this gene, thereby increasing the ABA content in tomato anthers and disrupting the formation of the pollen wall, resulting in pollen abortion (Yang et al., 2021).

m^6^A modification is also involved in the response of plants to heavy metal stress. When soybean plants are exposed to lead, the root growth is inhibited, while the transcriptome range of the m^6^A peaks increases (Zhang et al., 2023b). Exposure of rice to cadmium leads to abnormal root development and altered m^6^A modification profiles (Cheng et al., 2021). Cadmium stress also leads to an increase in the level of m^6^A modification across the soybean transcriptome (Han et al., 2023b). A recent study has shown that m^6^A modification is also involved in the copper stress response in *Arabidopsis thaliana* (Sharma et al., 2024).

#### Biotic environmental signals

**Figure 4:**
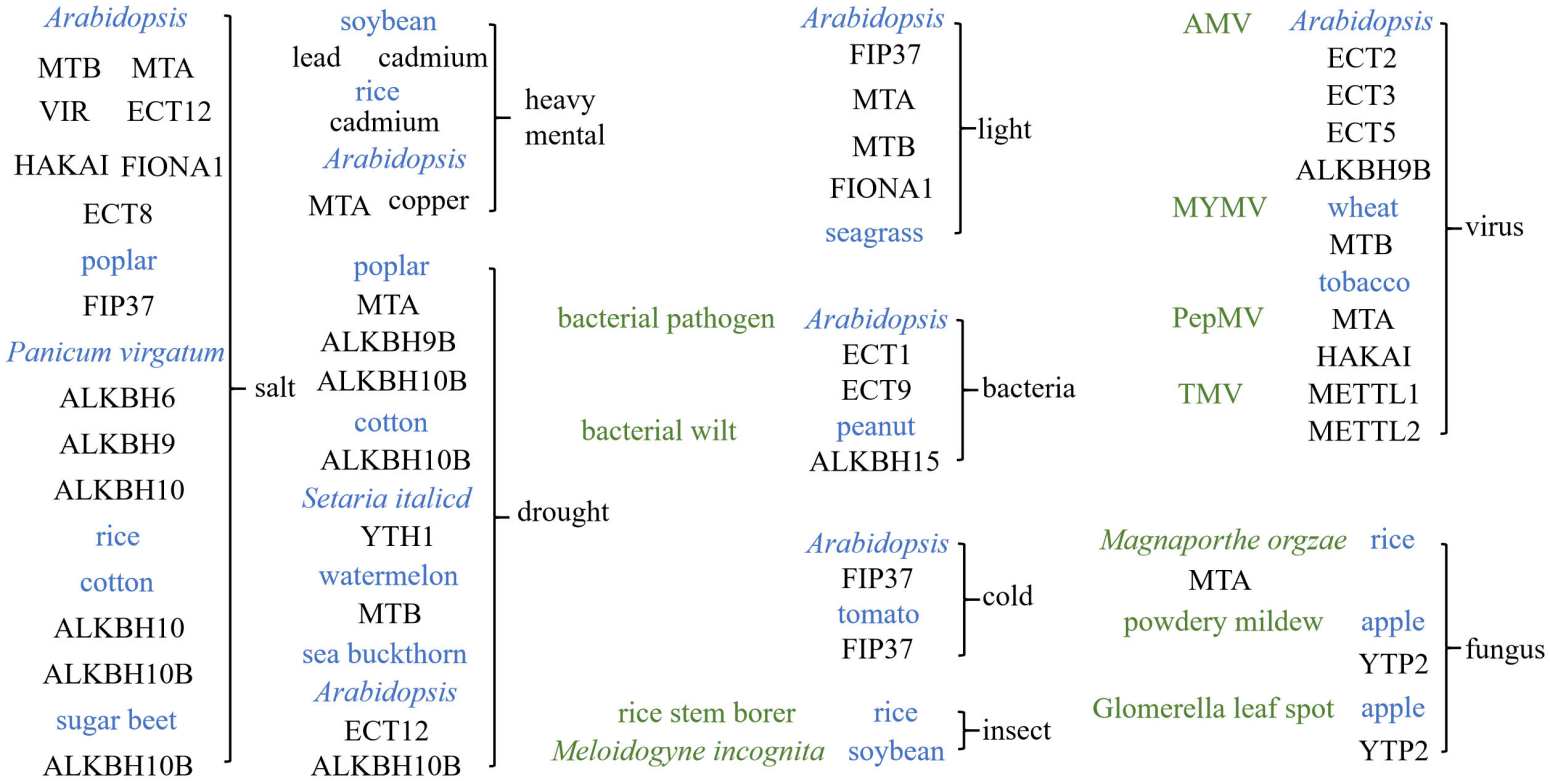
Function of *N* ^6^-methyladenosine (m^6^A) in the plant response to environmental signals. The effects of environmental signaling in various species under m^6^A regulation in plants include: salt signaling, heavy metal signaling, drought signaling, light signaling, cold signaling, bacterial signaling, insect signaling, viral signaling, and fungal signaling. The *blue color* represents the species of the plant, while the *green color* represents the specific biological stresses.

m^6^A modification has both negative and positive impacts on the responses of plants to external biological signals. It can affect the invasion of other organisms into plants. In *Arabidopsis*, the m^6^A demethylase ALKBH9B accumulates in the cytoplasmic granules and interacts with the coat protein of the Alfalfa mosaic virus (AMV), thereby positively regulating AMV infection. The inactivation of AtALKBH9B does not affect the stability of AMV particles, but blocks the virus from infecting plants through the epidermis. Inactivating ECT2/ECT3/ECT5 can restore the infectivity of AMV in partially resistant *alkbh9b* mutants (Martínez-Pérez et al., 2023). Moreover, ECT1 antagonizes the salicylic acid (SA)-mediated plant responses and can be recruited to ECT9 condensates, playing a negative role in plant immunity (Wang et al., 2023a; Lee et al., 2024). The wheat gene *TaMTB* is a disease susceptibility gene localized in the nucleus. *TaMTB* can bind to wheat yellow mosaic virus (WYMV) and upregulate its m^6^A levels, stabilizing the viral RNA and facilitating its transport to cytoplasmic bodies, thereby positively promoting viral infection (Zhang et al., 2021d, 2022b). Deficiency of MTA1 in the rice pathogen *Magnaporthe oryzae* reduced the appressorial penetration and invasive growth of *M. oryzae* and disrupted autophagy processes (Ren et al., 2022).

m^6^A modification can also regulate the immune capacity of plants. During viral infection of rice, different m^6^A peak distributions were detected on the same gene, which may contribute to different antiviral modes between different virus infections. In apples, overexpressing the reader gene *MhYTP2* can degrade the disease susceptibility genes *MdMLO19* and *MdMLO19-X1*, increase the translation efficiency of the antioxidant gene *MdGDH1L*, and enhance the resistance of apples to powdery mildew (Guo et al., 2022). MhYTP2 negatively modulates the resistance of apples to Glomerella leaf spot by binding to and degrading the *MdRGA2L* mRNA (Guo et al., 2023). Studies have shown that HAKAI and MTA increase the m^6^A modification of the Pepino mosaic virus (PepMV) RNA in *Nicotiana benthamiana* and tomato, suppressing viral invasion. In addition, the nonsense-mediated mRNA decay (NMD) factors UPF3/SMG7 can recognize the m^6^A-modified viral RNA complexes and limit plant viral infection by degrading viral RNA (He et al., 2023). In *N. benthamiana*, the overexpression of the METTL homologs *NbMETTL1* and *NbMETTL2* led to increased m^6^A modification levels and reduced tobacco mosaic virus infectivity (Yue et al., 2023). It is interesting that plant viruses could act as inducers to disrupt m^6^A methylation. The virus encodes the AlkB protein to promote virus infection (Yue et al., 2022). Moreover,AhALKBH15 led to a reduction in m^6^A and the upregulation of the level of the resistance gene *AhCQ2G6Y*, promoting bacterial wilt (BW) resistance in peanut (Zhao et al., 2024a).

m^6^A modification is also involved in the regulation of plant resistance to herbivores. Studies have shown that the overall m^6^A methylation levels are elevated in soybean under *Meloidogyne incognita* infection (Han et al., 2022). m^6^A modification also acts as the main regulatory strategy for the expression of the genes involved in plant–insect interactions, which is attributed to responses to rice stem borer (RSB) infestation (Li et al., 2024) (**Figure 4**).

## Discussion and prospects of m^6^A research

In recent years, there has been a great deal of research on the modification of m^6^A in animals, with research in plants following closely. With the continuous development of sequencing technologies and in-depth studies, researchers have revealed the important roles of m^6^A modification in plant RNA metabolism. However, there are still many unknown functions waiting to be explored. This article systematically reviews the sequencing techniques for m^6^A modification, its distribution characteristics in plants, the related components, and its functions, helping researchers gain a deeper understanding of how m^6^A plays a role in RNA epigenetic regulation in plants and providing new perspectives for future research.

With advances in technology, the elucidation of the mechanism of m^6^A modification will become clearer in future studies. The initial m^6^A-seq technique had issues with high sample demand, low resolution, and inability to quantify. However, the more recent techniques such as m^6^A-SAC-seq, GLORI, scDART-seq, and scm^6^A-seq have been optimized in terms of resolution and quantification. Although most of the current studies still utilize the m^6^A-seq technique, better alternatives are certain to emerge as technology continues to progress. m^6^A modification is widespread at different locations in RNA, dynamically regulating RNA metabolism by adding or removing modifications and exerting special functions (Shi et al., 2019). In current research, m^6^A modification generally functions by affecting mRNA metabolism. m^6^A modification influences seed germination, root growth, floral morphogenesis, plant height, fruit ripening, and senescence during plant growth and development (Lu et al., 2020; Tang et al., 2021; Rudy et al., 2022; Xu et al., 2022; Hu et al., 2022a; Xu et al., 2023; Cai et al., 2023ba; Sheikh et al., 2024). In addition, it plays a role in the responses of plants to abiotic environmental signals such as drought, cold, and heavy metal stress (Lu et al., 2020; Cheng et al., 2021; Vicente et al., 2023; Zhang et al., 2024). Furthermore, during plant viral infection, m^6^A modification may exhibit positive or negative functions (Cheng et al., 2021; Martínez-Pérez et al., 2023; Wang et al., 2023b; Yao et al., 2023b).

LLPS plays a role in many aspects of organisms, such as gene expression regulation, cell division, and stress response. In recent years, the field of LLPS has become a hot topic in the field of life sciences, and a series of important progresses has been made in plants (Jung et al., 2020; Liu et al., 2024). LLPS also plays a key regulatory role in the biological functions involving m^6^A, such as blue light signal transduction, chlorophyll homeostasis, and mRNA stability (Song et al., 2021; Wang et al., 2021; Lee et al., 2022; Jiang et al., 2023; Cai et al., 2024b). Follow-up studies can focus on the upstream regulation mechanisms of m^6^A in relation to LLPS.

The current findings suggest that m^6^A plays a regulatory role in the response of plants to environmental signals; however, the precise mechanisms remain elusive, leaving numerous unexplored territories. By elucidating the functions of m^6^A modification, we discover that it significantly impacts plant growth and development. This implies that by studying the modification of m^6^A in plants, we can anticipate its potential to enhance crop yield and stress resistance traits, thus providing valuable insights for future molecular breeding endeavors.

## Author contributions

YX: Writing – original draft. DZ: Writing – review & editing. LL: Writing – review & editing. Y-XX: Writing – review & editing. C-YZ: Writing – review & editing. Q-FM: Writing – review & editing. JW: Writing – review & editing. X-LT: Writing – review & editing. Y-LL: Funding acquisition, Supervision, Writing – review & editing.
